# Le sarcome du chorion cytogène: aspects cliniques et radiologiques

**DOI:** 10.11604/pamj.2020.37.301.23131

**Published:** 2020-12-02

**Authors:** Ahmed Hajji, Houda El Mhabrech, Manel Njim, Amira Daldoul, Awatef Hajjaji, Raja Faleh

**Affiliations:** 1 Service de Gynécologie Obstétrique, Centre de Maternité et Néonatologie de Monastir, Centre Hospitalier Universitaire Fattouma Bourguiba Monastir, Université de Monastir, Monastir, Tunisie,; 2 Service d´Imagerie Médicale, Hôpital Hadj Ali Soua, Ksar Hellal, Université de Monastir, Monastir, Tunisie,; 3 Service d´Anatomopathologie, Centre Hospitalier Universitaire Fattouma Bourguiba Monastir, Université de Monastir, Monastir, Tunisie,; 4 Service d´Oncologie Médicale, Centre Hospitalier Universitaire Fattouma Bourguiba Monastir, Université de Monastir, Monastir, Tunisie

**Keywords:** Sarcome, stroma endométrial, imagerie par résonance magnétique, chirurgie, radiothérapie, Sarcoma, endometrial stroma, magnetic resonance imaging, surgery, radiotherapy

## Abstract

Les sarcomes du stroma endométrial sont des tumeurs rares de la femme jeune. Le diagnostic est posé le plus souvent en post opératoire. Nous rapportons le cas d´une jeune fille de 22 ans admise dans un tableau de douleurs pelviennes avec métrorragies. L´imagerie a mis en évidence une masse utérine hétérogène. Le diagnostic de fibrome atypique était évoqué. Le diagnostic final est apporté par l´examen anatomopathologique de la pièce opératoire.

## Abstract (en)

Endometrial stromal sarcomas are rare tumors affecting young women. Diagnosis is most often made after surgery. We here report the case of a 22-year-old girl admitted with pelvic pain associated with metrorrhagias. Imaging objectified heterogeneous uterine mass. The diagnosis of atypical fibroid was suspected. Definitive diagnosis was based on histological examination of the surgical specimen.

## Introduction

Les sarcomes du stroma endométrial (SSE) sont des tumeurs rares et représentent entre 0,2 et 0,4% des tumeurs malignes de l´utérus et entre 7 et 15% des sarcomes utérins [1, 2]. Elles touchent surtout les femmes âgées entre 40 et 55 ans [1-3]. Le tableau clinique peut se révéler par des douleurs pelviennes et des métrorragies à un stade avancé mais souvent non spécifique. L´imagerie permet parfois d´évoquer le diagnostic sans pour autant le confirmer [3]. Le traitement consensuel des sarcomes localisés reste la chirurgie, le traitement adjuvant n´est pas encore clairement défini. Les principaux facteurs pronostics sont le statut hormonal de la patiente, le stade clinique, le type et le grade histologique, et la qualité de l´exérèse chirurgicale [4, 5].

## Patient et observation

**Figure 1:**
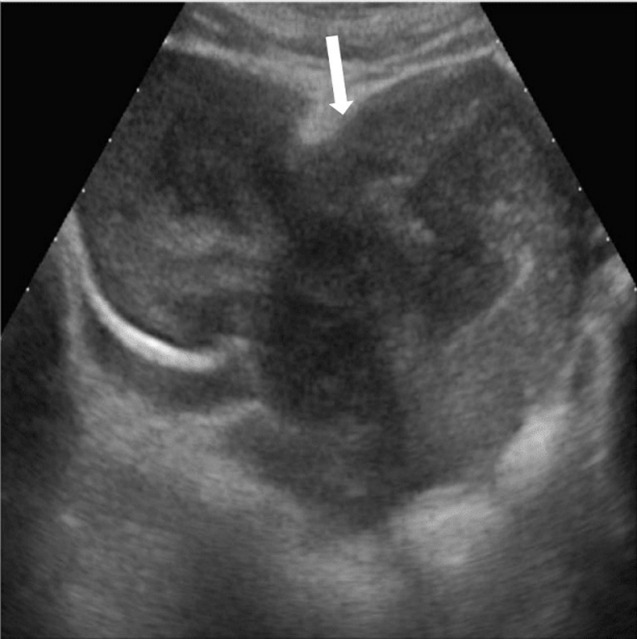
échographie pelvienne par voie sus-pubienne: utérus globuleux, d´échostructure hétérogène laissant émaner à partir du mur antérieur une masse arrondie bien limitée, d´échostructure hétérogène siège d´une zone centrale hypoéchogène

**Figure 2:**
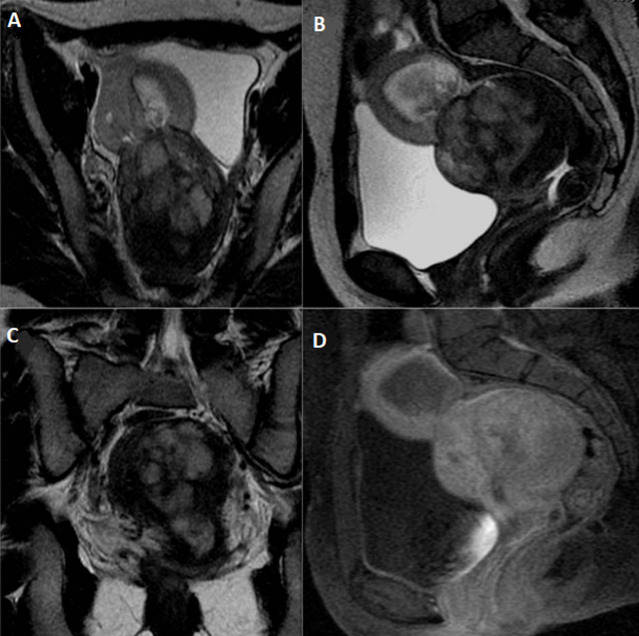
IRM pelvienne en coupes axiales (A), sagittale (B), coronale (C) en T2 et sagittale (D) en T1 Fat Sat après injection de Gadolinium: utérus augmenté de taille, de signal hétérogène

Nous rapportons le cas d´une jeune fille âgée de 22 ans, hospitalisée pour des douleurs pelviennes et des métrorragies. La patiente était opérée 6 mois auparavant pour un fibrome utérin corporéal antérieur de 7cm, on ne dispose pas du résultat de l´étude anatomopathologique de la pièce opératoire. L´examen clinique avait trouvé une patiente apyrétique avec une pâleur cutanéomuqueuse et une anémie à la biologie. L´échographie pelvienne avait montré une masse hétérogène et bilobée du fond utérin avec extension myométriale (Figure 1). Un complément par l'imagerie par résonance magnétique (IRM) pelvienne avait confirmé les données de l´échographie en montrant une masse bilobée et hétérogène du dôme utérin de 12cm, infiltrant le myomètre, siège de quelques calcifications, se rehaussant de façon hétérogène après injection de Gadolinium et renfermant une large plage nécrotique, non rehaussée (Figure 2). Le diagnostic d´un fibrome en dégénérescence hydro-myxoïde était le plus probable devant l´âge jeune et l´antécédent de myomectomie, mais une tumeur utérine agressive type sarcome n´était pas éliminée. Une tomodensitométrie (TDM) abdomino-pelvienne avait objectivé une volumineuse masse kystique hétérogène intra utérine sans signes d´infiltration locorégionale et sans adénopathies profondes ou lésions hépatiques avec absence de lésions d´allure secondaire tant à l´étage thoracique qu´abdominal. Le dosage des marqueurs tumoraux (antigène carcino-embryonnaire (ACE) et alphafœto-protéine (AFP)) était normal.

**Figure 3:**
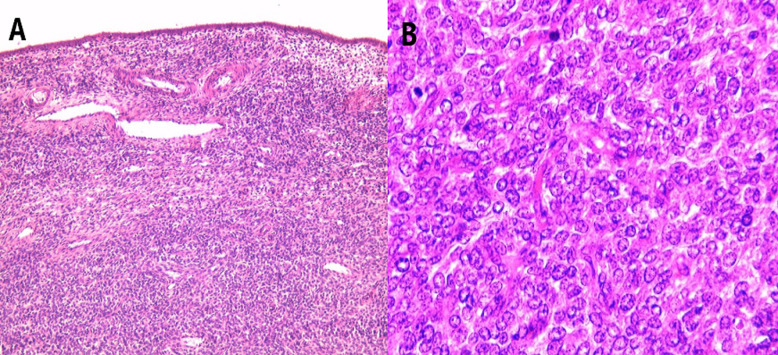
aspect anatomopathologique: la paroi utérine est infiltrée par des petites cellules bleues avec un cytoplasme peu abondant et une faible activité mitotique (A: HE x 200, B: HE x 400)

Un traitement chirurgical était décidé en réunion de concertation pluridisciplinaire. La patiente a eu une énucléation de la masse tumorale avec examen extemporané de la pièce opératoire qui révélait un sarcome stromal utérin, un complément d´hystérectomie totale avec annexectomie bilatérale était réalisé au même temps opératoire. Le résultat définitif d´anatomopathologie confirmait le diagnostic du sarcome du chorion cytogène de bas grade du corps utérin (SUCC) aux dépens de la paroi postérieur qui rompt la séreuse et réalise une masse extra utérine de 10cm sans atteinte annexielle (Figure 3). Le complément d´étude immunohistochimique avait montré une surexpression des récepteurs hormonaux (RE+/RP+). Le bilan d´extension était négatif (radiographie du thorax, radiographie du rachis et du bassin, échographie hépatique et scanner thoraco-abdomino-pelvien). Un traitement adjuvant comportant une radiothérapie et une hormonothérapie a été réalisé avec une évolution ultérieure favorable.

## Discussion

Les SUCC sont des tumeurs malignes d´origine mésodermique constituant le groupe de sarcomes ayant le meilleur pronostic avec un taux de survie à 10 ans estimé de 89% à 98% pour les stades I [2, 3]. Le diagnostic de ces tumeurs doit être évoqué devant toute augmentation rapide du volume du myome surtout chez une patiente ménopausée, une tumeur récidivante plusieurs fois au bout d´un intervalle court de temps ou une tumeur nécrosée accouchée par le col récidivant [4]. L´imagerie est peu spécifique. A l´échographie, l´aspect de fibrome remanié est le plus souvent rencontré. La tomodensitométrie est de même peu spécifique et ne distingue pas le sarcome utérin du fibrome en nécrobiose [2]. Elle garde sa place dans l´évaluation du bilan d´extension locorégionale.

Actuellement, avec l´avènement des séquences fonctionnelles et dynamiques après injection de gadolinium, l´IRM peut aider au diagnostic lorsque l´imagerie classique (échographie ou scanner) évoque une masse utérine atypique [2-4]. La masse est habituellement polyploïde et nodulaire, hypo intense en séquences T1 et présentant un signal hétérogène en séquences T2 avec des contours en hypo signal [3, 6, 7]. Elle se rehausse précocement après injection de Gadolinium avec une intensité de prise de contraste supérieure à celle du myomètre. L´existence de plages de nécrose intra lésionnelles est également très spécifique [2, 6]. Les séquences morphologiques T2 permettent de préciser l´extension myométriale par l´analyse de la zone jonctionnelle ainsi que l´extension à la séreuse utérine [7]. L´IRM permet donc de préciser l´extension locorégionale de la masse contribuant ainsi à la classification de la Fédération Internationale des Gynécologues Obstétriciens (FIGO) [8]. Comparativement au cancer de l´endomètre, le SSE est plus volumineux, présentant des contours plus irréguliers avec une extension myométriale de type nodulaire et se rehaussant plus intensément par le Gadolinium [6, 7]. L´atteinte ganglionnaire est présente dans presque 10% des cas [3, 7]. Dans notre observation, la masse présente un signal hétérogène en T2 occupant la totalité du corps utérin et présentant une extension exo-utérine à centre nécrotique. Elle se rehausse après injection de gadolinium délimitant de larges plages de nécrose.

La chirurgie constitue le premier temps de prise en charge des sarcomes utérins. L´intervention chirurgicale de référence est l´hystérectomie avec annexectomie bilatérale en cas de tumeur a priori limitée au corps utérin [2]. L´omentectomie et la lymphadénectomie doivent être pratiquées lorsqu´il existe des lésions suspectes sur l´épiploon et/ou une adénomégalie découvertes lors de l´exploration chirurgicale [2]. Cette exérèse devra être faite sans morcellement tumoral [9]. Dans les stades plus avancé (atteinte du rectum ou de la vessie, atteinte péritonéale, métastase à distance), la chirurgie est discutée. Néanmoins, l´hystérectomie avec annexectomie bilatérale semble être indiquée lorsqu´elle est techniquement réalisable [9].

Le traitement adjuvant est encore discuté. La plupart des auteurs s´accordent sur le fait que l´irradiation adjuvante apporte un bénéfice en termes de contrôle local de la maladie même si le bénéfice sur la survie n´est pas certain. Une diminution du nombre de récidives pelviennes, peut justifier la prescription d´une radiothérapie adjuvante. Ce bénéfice est souvent attendu pour les tumeurs de haut grade histologique [8, 9]. Dans notre cas, la patiente a bénéficié d´une radiothérapie adjuvante à la dose de 50 grays suivis d´une hormonothérapie. Quant à la chimiothérapie, son intérêt demeure incertain, bien qu´elle soit probablement à recommander dans des situations précises (patientes jeunes, stade FIGO élevé, grade histologique élevé). Elle ne doit cependant pas trop retarder l´irradiation [5, 9]. Le pronostic des sarcomes utérins reste jusqu´à nos jours mauvais, mais meilleur que les autres types de sarcomes utérins. Les principaux facteurs rapportés étaient le statut hormonal, le type histologique, le grade histologique, le stade clinique et la présence d´un résidu tumoral après chirurgie [4, 5].

## Conclusion

Les sarcomes du stroma endométrial (SSE) est une maladie rare, son diagnostic doit être précoce car la survie des patientes est corrélée au stade tumoral. Paradoxalement ce diagnostic est orienté par les données cliniques et radiologiques et confirmé le plus souvent sur la pièce d´hystérectomie.
